# Exploring national human resource profile and trends of Prosthetists/Orthotists in South Africa from 2002 to 2018

**DOI:** 10.1080/16549716.2020.1792192

**Published:** 2020-07-29

**Authors:** Luphiwo Mduzana, Ritika Tiwari, Ned Lieketseng, Usuf Chikte

**Affiliations:** a Department of Rehabilitation Medicine, Faculty of Health Sciences, Walter Sisulu University, Mthatha, South Africa; b Division of Health Systems and Public Health, Department of Global Health, Stellenbosch University, Stellenbosch, South Africa; c Centre for Rehabilitation Studies, Department of Global Health, Stellenbosch University, Stellenbosch, South Africa

**Keywords:** Demographics, Prosthetists/Orthotists, human resources, rehabilitation, South Africa

## Abstract

**Background:**

The World Health Organization (WHO) in 2017 estimated that around 35–40 million people require prosthetic or orthotic services. The Framework and Strategy for Disability and Rehabilitation 2015–2030 for South Africa highlights a shortage of human resources for disability and rehabilitation services to manage the various risks and types of impairments faced by the population.

**Objective:**

To describe the demographic trends of Prosthetists/Orthotists (P/O) registered with the Health Professions Council of South Africa (HPCSA) from 2002 to 2018.

**Methods:**

The study was a retrospective record-based review of the Health Professions Council of South Africa (HPCSA) database from 2002 until 2018. The database of registered Prosthetists/Orthotists was obtained from the HPCSA.

**Results:**

Data were analysed using the Statistical Package for the Social Sciences (SPSS version 22.0). In 2018, there were 544 P/Os registered with the HPCSA with a ratio of 0.09 P/Os per 10,000 population. There has been an average annual increase of 6% from 2002 to 2018. The majority (71.9%) of P/Os are located in the more densely populated and urbanized provinces, namely Gauteng, KwaZulu-Natal and Western Cape. The majority of registered P/Os identified as white (61%) followed by Black (22%), Indian (7%) and Coloured (2%). Most of registered P/Os are under the age of 40 years (54.2%) and males make up 73% of the registered P/Os.

**Conclusion:**

This study highlights the unequal spatial distribution trends of P/Os which could be accounted for by South Africa’s apartheid history and the subsequent slow pace of transformation. Addressing the existing shortages is necessary to expand access to P/Os services and to ensure the motivation, planning and provision of adequate infrastructure to provide these services. The study presents a compelling case for the prioritization and strengthening of this workforce for the achievement of effective universal health coverage for persons with disabilities.

## Background

Achievement of Universal Health Coverrage (UHC) requires a well-trained workforce not only to drive the health care system but to also provide appropriate health care to all. Worldwide, about 15% of the population with majority located in the Global South, experience some type of disability and are in need of rehabilitation services [1]. The inadequacy of the current rehabilitation workforce is also noted by the World Report on Disability as a global issue [1]. The supply and need for human resources of health related rehabilitation services reported in a global study showed that 92% of the burden of disease ‘years of life lost’ require rehabilation support and assistance [2]. Yet, rehabilitation services are largely unavailable for persons with disabilities globally with many countries not referring to rehabilitation in national planning and reviews of reseources for health [1]. The United Nations report on the realization of Sustainable Development Goals (SDGs) by 2030, suggests that approximately 64% of persons with disabilities who need rehabilitation are unable to access services [3]. Inadequate health and rehabilitation services for people with disabilities highlight the urgent need for improvement at this front to realize the goals of health and well-being as articulated in SDG 3.

Strengthening access to rehabilitation services includes addressing human resources which are a neglected component of health systems development and are often absent from national health sector plans or human resources for health (HRH) development strategies [2,4–6]. Rehabilitation services are provided by a diverse range of professional, technical, and mid-level workers located in the public, private and NGO sector. A core rehabilitation human resource team includes occupational therapists, speech and language therapists, audiologists, physiotherapists, prosthetists and orthotists, and the mid-level workers allied to these fields. Other professions playing significant roles include psychologists, social workers, podiatrists, and orientation and mobility. While there is an unmet need for rehabilitation in all the aforementioned specialized rehabilitation professional categories [7], this paper focuses on prosthetists and orthotists given the paucity of research in these categories in South Africa.

The WHO standards for prosthetics and orthotics [7] suggests that at least five to ten prosthetic and orthotic professionals are required for a population of a million. While higher-income countries usually have 15 to 20 or more Prosthetist/Orthotists (P/Os) per million population, lower income countries usually have as low as one per million population and this hinders adequate service provision of appropriate quality to the population. Current evidence from the International Society for Prosthetics and Orthotics (ISPO), however, shows that in some high-income countries, the number of registered prosthetists, orthotists, technicians and technologists does not reach the minimum number of required professionals. In the African, South-East Asia and Western Pacific regions, the number of practicing professionals is only one tenth of the number required [8].

Recently, the World Health Organization (WHO) and the World Bank estimated that while there is 35–40 million people currently requiring prosthetic or orthotic services, only 1 in 10 persons has access to such services [7]. With a significant rise in aging populations globally as well as an increase in the incidence and prevalence of non-communicable diseases such as diabetes and a concomitant need for rehabilitation, more than 2 billion people are projected to require prosthetic and/or orthotic treatment by 2050 [9]. These statistics underline the growing current unmet need and highlight the likelihood of significant underservicing of those who require prosthetic and/or orthotic care [4]. The picture is similar to what is experienced in the South African context and compounded by a sharp increase in the number of lower limb amputations performed due to the consequences of increasing incidences of Type 2 diabetes mellitus [10,11]. This provides an indication of the demand for pre-and post-prosthetic rehabilitation to prevent poor physical and psychological outcomes amongst people with lower limb amputations. An increasing number of people will continue to require rehabilitation in primary healthcare settings to access prosthetic and orthotic services which will continue to escalate [12].

The World Health Organization Report on access to rehabilitation in primary health care settings acknowledges that a stronger rehabilitation workforce capacity may make rehabilitation more accessible especially at primary care levels [12]. Other factors hampering access to prosthetic and orthotic services include a broad lack of understanding of the benefits and need for these services as well as an infrastructural failure to provide appropriate services [8]. The availability of an adequate workforce and resources, including appropriate assistive technology, are integral to improved rehabilitation access and successful integration of rehabilitation at all levels of healthcare. In South Africa, despite the fact that the category of Prosthetics and Orthotics was established in 1947, the profession still faces serious shortages of staff country-wide.

Prosthetists/Orthotists defined as ‘a health care professional who uses evidence-based practice to provide clinical assessment, prescription, technical design, and fabrication of prosthetic and/or orthotic devices, work independently or as part of the health professional team. They set goals and establish rehabilitation plans that include prosthetic/orthotic services and clinical outcome measures. The profession aims to enable service recipients so that they have equal opportunities to fully participate in society’ [8]. For the purpose of this paper, we use the term Prosthetist/Orthotists as per the ISPO education standards for P/O occupations. However, it is worth noting that South Africa still uses the terminology ‘Medical Orthotist and Prosthetists’ under the Health Professions Council of South Africa which dictates the nomenclature to the profession. This terminology has been in existence since the first registration of the profession in 1989. Consequently, there is currently no differentiation between degree and diploma holders in terms of occupation title and everyone is deemed to register as a Medical Orthotist and Prosthetist as affirmed by the Government Gazette 31,535 of 31 October 2008. The power to changing the terminology to align with the international terminologies depends hugely on the two existing local associations (MOPASA and SAOPA). The revised scope of practice, however, corresponds to what the international standards refer to as P/Os, despite having retained the old terminology. In South Africa, since 1985 and up until 2003, the highest qualification a P/O could attain was a diploma certificate [13]. The Bachelor of Technology (BTech) qualification in Medical Orthotics and Prosthetics was introduced in 2003 and two universities have introduced a Bachelor of Science (BSc) degree in Medical Prosthetics and Orthotics. Currently, South Africa has three universities that offer a qualification in Medical Prosthetics and Orthotics namely Tshwane University of Technology (TUT), Walter Sisulu University (WSU), and Durban University Of Technology. A graduate in Medical Orthotics and Prosthetics (BSc: MOP) program is capable of practicing as a clinician, developing and managing a clinic/laboratory or providing services as a private practitioner [14]. These professionals have to be registered with HPCSA under the Occupational Therapy, Medical Orthotics and Prosthetics and Arts Therapy Board in accordance with the Health Professions Act No. 56 of 1974, to practice and provide services through both private and public sectors. There is, however, no clear HPCSA data indicating the distribution between the two sectors. Data obtained from the South African Orthotic and Prosthetic Association (SAOPA) in 2017 showed availability of 182 registered P/Os servicing the private sector. The Provincial Department of Health has identified a huge demand for P/Os and their services in the Provinces within South Africa [14].

The 2012 South African report on human resource document make no mention of P/O human resources in health workforce planning [15]. The unavailability of such data continues to place this profession receiving little attention which may lead to limited funding, as these services are frequently not budgeted for in national health and social insurance systems. In 2015, the Framework for Disability and Rehabilitation 2015–2030 [16] was developed in South Africa with an intention to integrate disability and rehabilitation services at all levels of the healthcare system. Though this Framework and Strategy for Disability and Rehabilitation (FSDR) 2015–2030 displays the vacancy numbers of other rehabilitation professionals’ country wide, data on Prosthetics and Orthotics professionals remains lacking. This strategy, however, acknowledges shortages and advocates for the need to develop and improve human resources for disability and rehabilitation services if we are to achieve universal health coverage and overcome existing inequities [16]. Recent findings from an audit of national health care facilities in SA reveal that only 6–20% of primary health care (PHC) facilities offered rehabilitation services [17]. Given that universal health coverage (UHC) is about making services more available, accessible and affordable, it may be argued that UHC cannot be attained without consideration of specific needs of persons with disabilities and better health services as the most vulnerable group [18].

There is a dearth of research of human resources on Medical Orthotists and Prosthetists human resources, particularly in South Africa. Specifically, no study in SA has profiled P/Os workforce and forecasted gaps. One of the bottlenecks for expanding access to rehabilitation services is shortages of appropriately trained and deployed human resources [19]. As such, a focus on human resources is integral to enhancing accessibility to rehabilitation services. Assessing availability and distribution of P/Os is needed to understand the scope of shortages as well as the capacity of the health system to meet health-related rehabilitation service objectives in South Africa. Such an understanding is imperative for policy-making which recognizes rehabilitation as integral to other critical aspects of healthcare. It is also particularly important to inform the planning, financing and implementation of the FSDR intentions. The primary aim of this study was to describe the changing demographic trends of orthotists and prosthetists registered with the Health Professions Council of South Africa (HPCSA) from 2002 to 2018 as a first step towards understanding the supply and status of human resources for Medical Orthotists and Prosthetists in South Africa.

## Methods

A retrospective record-based review of the HPCSA database from 2002 until 2018 was conducted. The database was procured by Department of Global Health, Stellenbosch University through a special written request to the HPCSA. This database includes information on all registered prosthetists and orthotists including age, gender, racial categories, location and category of practice. The HPCSA was established in 1974 as a statutory body under the Health Professions Act 56 of 1974, has 12 Professional Boards under its ambit, and controls the education, training and registration for practicing of health professions registered under the Health Professions Act [20]. HPCSA collects data electronically through online registration and renewal of registration process requires a valid practice number, ID, address and password. HPCSA ensures safekeeping of data [21,22]. The deidentified data was obtained from a written request and permission granted to UC. These data were accessed, analysed and handled by RT. The data were stored in an MS Excel document in a password protected computer. The HPCSA data include only factual information substantiated by the health practitioners registering with them, and is presented in an unbiased manner [22]. However, HPCSA does not provide whether the professional is located in the public or private sector (or both), emigration, death and retirement details [23].

A similar approach was adopted in a previous study [18] and collected relevant data using a data collection sheet with the following variables included: (i) category of health personnel, (ii) category of practice, (iii) geographical location, (iv) population category and (v) gender. In this paper, we have used the term population along the lines of the Population Registration Act (Act No. 30 of 1950) which classified South African citizens into four major categories namely ‘white’, ‘coloured’ ‘indian’ and ‘black’ based on the colour of their skin [24]. Although the legislation was repealed in 1991, in some instances it is still required to report along these categories in sectors such as the Department of Higher Education and other employment sectors. It is also used as a measure to monitor the redress in the education and training of orthotists and prosthetists who were previously denied access to such training in terms of legislation.

The dataset was accessed, collected and analysed by LM and RT and accuracy was cross-checked by LN. Data were entered into a Microsoft Excel spreadsheet and analysed using the Statistical Package for the Social Sciences (SPSS version 22.0). Frequency distributions, cross-tabulations and graphical representations were used as descriptive statistical methods. Anonymity and confidentiality of all personnel was ensured as the data accessed from the HPCSA and presented in this paper is de-identified. Ethical approval and a request for waiver of informed consent for this retrospective study was obtained from the Stellenbosch University Health Research Ethics Committee (HREC Reference No: X19/10/039)

## Results

### Profile of Prosthetists/Orthotists

**Figure 1.**
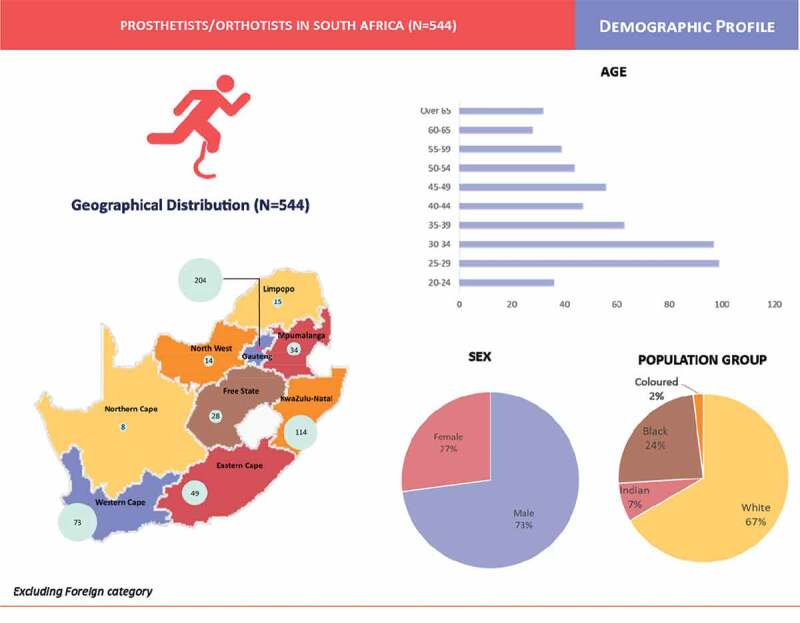
Profile of Prosthetists/Orthotists.

A total of 544 P/Os was registered with HPCSA in 2018 with females comprising 27% compared to 73% of males. Figure 1 provides a summary of the data according to geographical distribution, age, provincial distribution, age, sex and population categories.

### Growth in the number of Prosthetists/Orthotists

**Figure 2.**
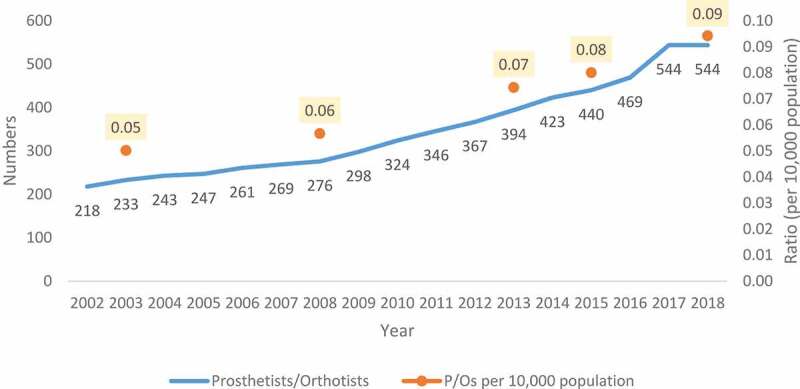
Number of Prosthetists/Orthotists registered from 2002 to 2018 and P/Os: Population ratios.

The number of P/Os has almost doubled from 2002 (N = 218) to 2018 (N = 544). with an average annual increase of 6% over the period. Both the SA population and number of P/Os increased over the 15-years period (Figure 2). Compared with a 24.3% increase in the South African population, the ratio of P/Os per 10 000 population in SA also increased from 0.05 in 2003 to 0.09 in 2018.

### Geographical distribution by province

The majority of P/Os are located in the more densely populated and urbanized provinces namely; Gauteng (n-204, 37.5%), KwaZulu Natal (n-114, 21.0%) and Western Cape (n-73, 13.4%). These were followed by Eastern Cape (n-49, 9.0%), Mpumalanga (n-34, 6.3%) and Free State (n-28, 5.1%). Limpopo (n-15, 2.8%), North West (n-14, 2.6%) and Northern Cape (N-8, 1.5%) hosted the lowest numbers of P/Os. (See Table 1).

**Table 1.** Geographical distribution of P/Os and comparison of trends from 2003.

		2018				2003		
	Category	Prosthetists/Orthotists	Percentage distribution of P/Os in the province (as a % of total number in SA)	Population of the province (as a % of total national population)	P/Os per 10,000 population	Prosthetists/Orthotists	P/Os per 10,000 population	Percentage change in MOPs per 10,000 population from 2003 to 2018
1	Gauteng	204	37.5	25.3	0.14	91	0.10	40.0
2	KwaZulu-Natal	114	21.0	19.6	0.10	35	0.04	150.0
3	Mpumalanga	34	6.3	7.9	0.08	11	0.03	166.7
4	Western Cape	73	13.4	11.5	0.11	34	0.07	57.1
5	Limpopo	15	2.8	10.2	0.03	7	0.01	200.0
6	Eastern Cape	49	9.0	11.5	0.08	27	0.04	100.0
7	North West	14	2.6	6.8	0.04	6	0.02	100.0
8	Free State	28	5.1	5.1	0.10	18	0.06	66.7
9	Northern Cape	8	1.5	2.1	0.07	3	0.03	133.3
10	Foreign	5	0.9	-	-	1	-	
	**TOTAL**	544	100	100	0.10	233	0.05	100.0

Eastern Cape and Western Cape have the similar sized populations, yet the Western Cape has higher density of P/Os per 10 000 population than Eastern Cape (1.4:1). Although North West has half the population of the Western Cape, the density of P/Os is one-sixth (2.6 per 10 000 population) of that of the Western Cape (13.4 per 10 000 population).

### Age distribution

In 2018, the majority of registered P/Os were between the ages 25 and 34 (36%), followed by the group between ages 35 and 50 years (30.5%). The trend of change in age distribution cannot be provided as the data provide age details of the registered professionals as on date and not at the time of registration.

### Population category of Prosthetists/Orthotists

**Figure 3.**
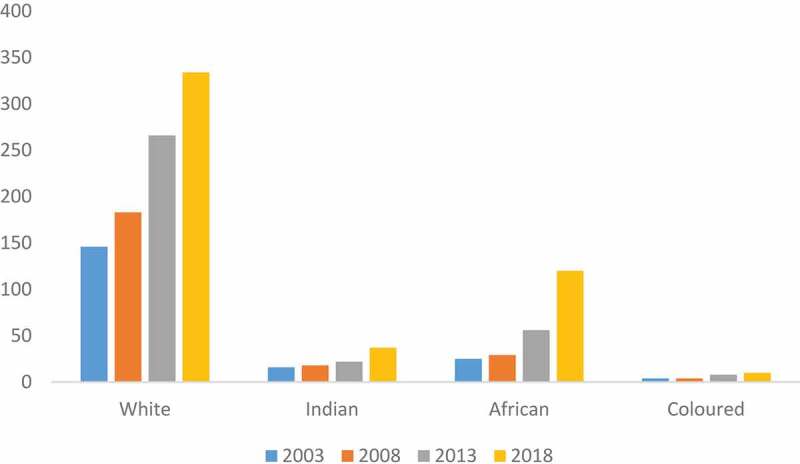
Distribution by population group over 5-year intervals.

In 2018, the profile of registered P/Os in South Africa who identified as white was 61%, followed by Black (22%), Indian (7%) and Coloured (2%) (See Figure 3).

The breakdown of registered P/Os for five-year intervals starting in 2003 shows growth in the number of P/Os across all population groups.

### Distribution by sector

There is no data on the public-private sector split available from the HPCSA database for P/Os.

### Distribution by sex

**Figure 4.**
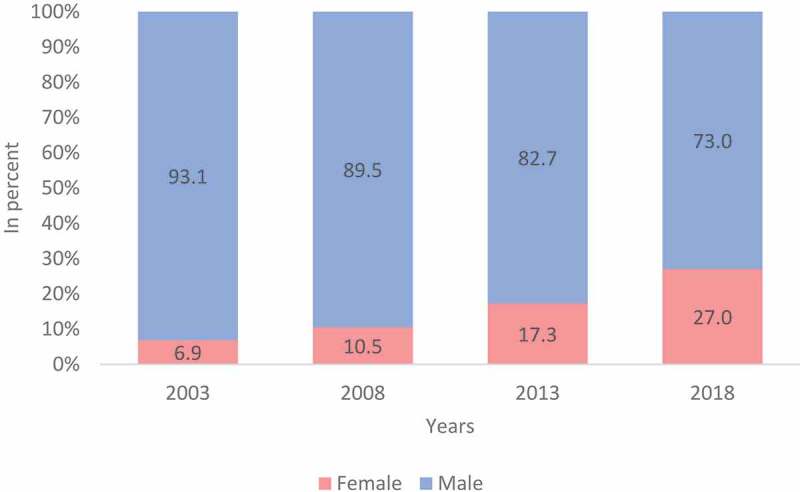
Distribution by sex over 5-year intervals.

The representation of the females has grown from 6.9% (2003) to 27% (2018). The percentage of male P/Os have decreased by 20% i.e. from 93.1% (2003) to 73% (2018). The gradual change in distribution of sex of P/Os has been represented in Figure 4.

### Demographic trends by population group, age and sex

**Figure 5.**
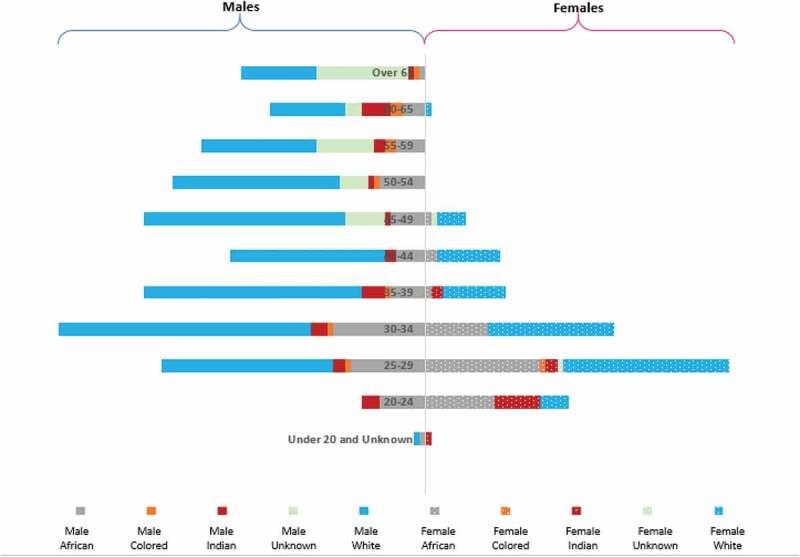
Breakdown of registered Prosthetists/Orthotists by population group, age and sex.

Tracking demographic variables of P/Os by age, population group and sex (see Figure 5) shows that in 2018 the P/Os workforce was predominantly comprised males and falling in the population group classified as white across all age groups. While the number of P/Os decreases with increasing age across all population groups. Interestingly, the females in the profession are mostly younger and categorized as White.

## Discussion

With a population of 59,308,690 in 2020, South Africa has a GINI coefficient of 0.63 and inequity remains one of the defining features of the economic, social and health system [25]. The health inequity is stark when comparing the population dependent on the public sector (84%) and those dependent on private sector (16%), as well as availability of services between, and within, urban and rural public sector users. Part of the vision for UHC is to facilitate delivery and access to quality services to all through the deployment of an adequate, well-trained workforce.

This study set out to describe the status of human resources for medical orthotists and prosthetists in South Africa. Under the jurisdiction of the HPCSA three professional categories are registered in the P/O register namely; the Medical Prosthetist/Orthotist, Assistant Medical Prosthetist/Orthotist and the Orthopaedic footwear technicians. The former consists of qualified professionals (both diploma and degree) who mainly drive the clinical service delivery and occupy leadership positions. While both these diploma and degree professionals competently perform clinical service, a majority are diploma holders and thus fall within the category of associate P/O in terms of the standards set by the international standards for occupations. This means that, there remains a shortage of what is classified as P/Os benchmarked against the ISPO standards. Degree training was recently introduced and only three universities offer such training. Both the assistants and technicians do not hold any formal qualification but are provided with in-house on-the-job training. These assistants and technicians both fall within the non-clinical category, however there is no formal higher education certification in the South African context as yet. Currently, there exists a draft of minimum standards for the training of orthopaedic technical assistants which will be translated into formal education to professionalize the assistant medical orthotist/prosthetist. Consistent with literature, these technicians are often under-represented in the workforce and have limited standardised training opportunities globally [8].

The profession and services rendered by the P/Os are relatively unknown not only in the health sector, but also by the people who could benefit from these services. The public is often unaware of their existence, what the services may provide and consequently may not demand them [7]. This study shows that majority of P/Os in South Africa are likely to be male and classified as white and in the age groups of 25–34 and 35–50 years. While growth in the number of P/Os was noted across all population groups, the breakdown showed the slow pace of transformation between population categories. For a number of years, within a racially segregated and discriminatory system of training, those classified as white could access the field of P/Os and this legacy is still evident in the demographic analysis of professionals registered within the P/O profession by population category (61% of P/Os are of classified as white). Similarly, the profession is overwhelmingly dominated by males (73%). The geographic distribution trends revealed across provinces in the 15-year period show that the three urbanized provinces (Gauteng 37.5%, KwaZulu- Natal 21% and Western Cape 13.4%) enjoy more human resources. Gauteng and Western Cape also have more P/Os in the private sector while the public sector continues to compete adversely as the workforce and skills migrate to the private sector. The Northern Cape (1.5%) has the lowest percentage of P/O human resources followed by North West (2.6%), Limpopo (2.8%), Free State (5.1%), Mpumalanga 6.3%, and the Eastern Cape with about 9.0% of the P/O human resources. These geographical, population group and gender differences can be accounted for by the country’s history

Historically, apartheid segregation in South Africa also influenced the rehabilitation workforce thus leading to an unequal distribution of this workforce not only spatially between the rural and urban developments but also who could access the training. The segregation was based on both on sex and population group in its design. While the segregatory and discriminatory legislation and policies with the selection of entering P/O students were abolished in 1994 and transformation efforts in the training universities are noted, the factors that impede the selection of a more diverse P/O workforce merit further investigation. An increase in human resources for P/Os services will not only expand service delivery but also ensure that adequate funding and infrastructure to provide these services are planned and motivated.

**Figure 6.**
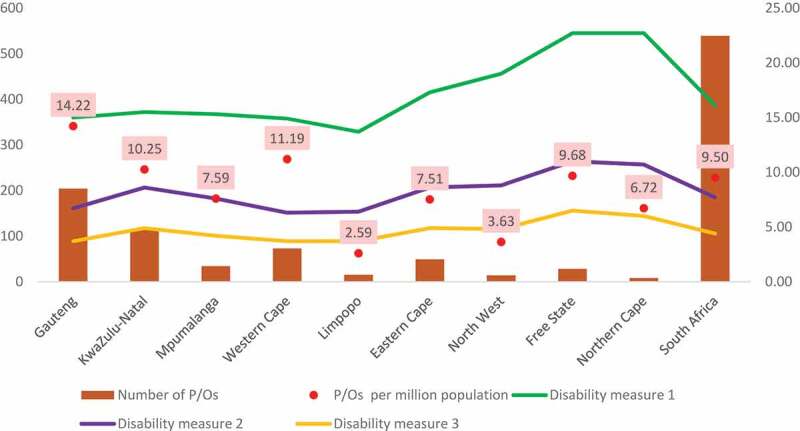
Comparison between availability of P/Os and disability prevalence.

South Africa currently has varying disability reported prevalance rates (4.4%, 7.7% and 16.1%) depending on which measure is used. The spatial distribution of the P/Os necessitates a closer analysis of the workforce supply against the disability prevalence rates. Stats SA uses three different measures to calculate disability prevalence in SA (reported in Figure 6 below). Disability measure 1 refers to the broad disability measure which includes all persons aged 5 years and older that reported ‘some difficulty’ in any of the domains of functioning, ‘a lot of difficulty’ and ‘cannot do at all’ to any of six domains of functioning. Disability measure 2 refers to the UN disability index which includes all persons aged 5 years and older that reported ‘some difficulty’ in at least 2 domains of functioning, ‘a lot of difficulty’ and ‘cannot do at all’ to any of six domains of functioning”. Disability measure 3 refers to the severe disability measure which includes all persons age 5 years and older that reported ‘a lot of difficulty’ and ‘unable to do at all’ to any of six domains of functioning (StatsSA 2018). The prevalence rates by province reveals that, in the 2016 community survey, persons with disabilities are located in greater numbers in non-urban areas compared to those in urban areas [26]. The similar trend is reflected with the 2011 census in SA. In contrast, P/Os workforce is more informs available in urban areas as compared to rural. When looking across the three measures, both the broad and severe disability measures showed a downward trend in disability prevalence in both urban and non-urban areas while the United Nations (UN) disability measure showed an increase in disability prevalence in urban areas (from 6.3% which was recorded in Census 2011 to 7.2% in Community Survey 2016). This information should inform decision making on the allocation of resources in underserved areas where rehabilitation services are needed for those who require prosthetic and/or orthotic care.

Stats SA, 2018. Community Survey 2016: Profiling socio economic status and living arrangement of persons with disabilities in South Africa.

Notably, an observation of the supply of professionals per province against the provincial disability prevalence rates as per the 2016 community survey (See Table 2 below) also shows that, within South African provinces with higher density of P/Os per million population such as Gauteng, Western Cape and KwaZulu Natal – the disability prevalence is lower. Other provinces with lesser density of P/Os per million population such as Free State, Eastern Cape and North West have higher disability prevalence. The same picture is seen when the analysis is done using the 2011 census data. A similar trend was found by Gupta et. al [2]. in a global analysis of the supply of human resources for health against selected causes of Years of life lost (YLL). This study suggested that countries with the highest burden of disability-related health conditions tend to be those with the lowest supply of rehabilitation workers. Similarly, when analysing the supply–need dynamics, countries with higher rehabilitation needs tend to have lower numbers of skilled rehabilitation workers.

**Table 2.** A plot of P/Os ratios and numbers per province against disability prevalence per province.

Province	P/Os per million population	Number of P/Os	2016 Community survey Disability measure 1	2016 Community survey Disability measure 2	2016 Community survey Disability measure 3
Gauteng	14.22	204	15	6.7	3.7
KwaZulu-Natal	10.25	114	15.5	8.6	4.9
Mpumalanga	7.59	34	15.3	7.6	4.2
Western Cape	11.19	73	14.9	6.3	3.7
Limpopo	2.59	15	13.7	6.4	3.7
Eastern Cape	7.51	49	17.3	8.6	4.9
North West	3.63	14	19	8.8	4.8
Free State	9.68	28	22.7	11	6.5
Northern Cape	6.72	8	22.7	10.7	6
**South Africa**	**9.50**	**539**	**16.1**	**7.7**	**4.4**

The comparison between population growth and that of P/Os in the country exposes serious shortages. Globally, lower supplies of rehabilitation professionals have been noted among low-middle-income countries particularly [2]. ISPO standards suggest a need for at least five prosthetic and orthotic professionals for over 1 million population. While the population increased by 24.3%, this study shows an increase in the ratio of P/Os from 0.05 to 0.09 per 10 000 population. This means that there are 9 P/Os per million population. However, within provinces with a higher disability prevalence, many of these professionals service the private sector which only caters for 16% of the South African population [6]. This highlights the shift to and preference for working in private sector which depletes the public sector rehabilitation workforce.

**Figure 7.**
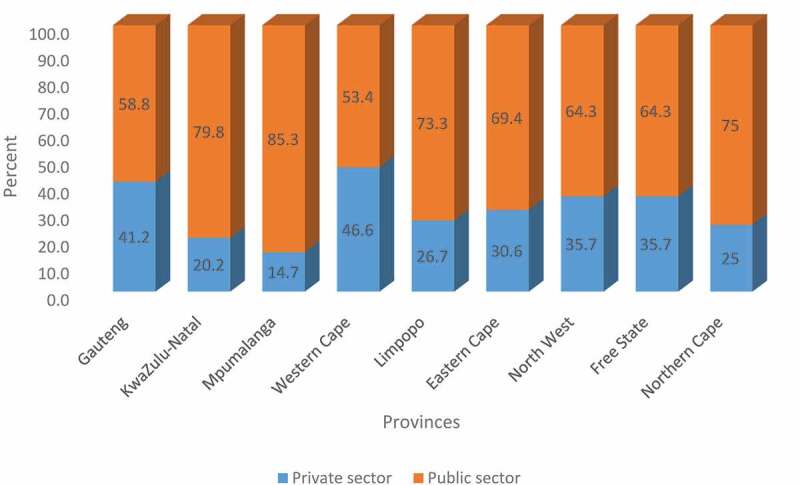
Percentage distribution of P/Os employed in public (based on HPCSA data) and private sectors based on 2017 SAOPA data by province.

Currently, both the private and public sectors in South Africa are not able to provide available data on human resource counts and demographics regularly. There is also no single repository that includes all the necessary information within each sector or nationally. The data captured by HPCSA as a regulatory body typically only consists of qualified professionals who have registered regardless of the nature of current work, activity, immigration status or geographical location in the country [2]. Other bodies such as Medical Orthotist Prosthetist Association of South Africa (MOPASA) organizing public sector employees and SAOPA catering for private sector employees also provide a source for HR data. If we use the SAOPA data, Figure 7 represents percentage distribution of P/Os in 2017 between public (based on HPCSA data) and private sectors by province. Such a depiction demonstrates the loss experienced by the public sector which services majority of the SA population. However, there are concerns about the accuracy of such data because these data sets are not always up to date, they consists of data of affiliated members only (based on voluntary membership) and often do not provide a view of which sector the registered professionals are located in the public or private sector. A similar struggle of data was noted in a global cross-national study of rehabilitation human resource with low-and-middle-income countries particularly reporting less on availability of human resources, a result of limited availability and use of quality, comparable data and information across countries [2]. This study also reported systematic differences in reporting linked to the nature of national data sources ranging from census to payroll data or professional bodies.

Globally, often data contained from the ministry of health only records its employees or posts [2]. In SA, the persal system can be used to identify those in public sector but current data does not have information on P/Os. Thus, differences are still found with regard to which rehabilitation-related occupational codes are disseminated. While SAOPA is the main data source for private sector, the database is also limited in its information with quality and collation issues, perhaps mainly because it is purely based on voluntary membership and it is not for regulatory purposes. In order to give a much more comprehensive picture of the workforce, one needs to gather data from these several organizations/bodies and datasets. These reality makes it difficult to regularly replicate and influence robust mapping and planning. Comparability also becomes difficult given that each database records differently and this translates to limitations with comprehensive monitoring of workforce trends. To accurately present a clearer picture, further comprehensive data is needed with additional variables such as, who is in which sector which includes public facilities, private facilities, community-based service delivery, academic training or research institutes. Data are also required professionals in actual practice, who are no longer practising, whether registrants are out of the country temporarily or permanently, what type of employment practitioners are located in among other factors. Nevertheless, we believe that this study provides much needed data on the current human resource status of P/Os in the country – information that could facilitate efforts towards improving future supply and demand as per the intentions of the FSDR.

Assuming the public sector user population to be the proportion of the population without health insurance, an extrapolation of current public-private split of P/Os on population projections up to 2030 [27] (maintaining current densities of P/Os) suggests that around 630 will be needed in public sector and 211 in private sector. Thus, it can be assumed that in next 13 years, an additional 91 personels will be needed to cater for the public sector needs and 29 for the private sector. No other rates from other countries were found to enable a comparison. This lack of data is a common issue as highlighted by ISPO (2018).

A related data issue is that of the assistive devices provided by P/Os or needed by the SA population. An observation from the 2018 Statistics SA report is that, on the question of assistive devices, there is no explicit question category for recording statistics for orthotic and prosthetic devices other than a space labelled ‘other devices’. Even under the broad category of mobility devices, there remains a need to accurately record orthotic and prosthetic devices. This may be problematic in terms of quality evidence that can be offered as it is argued by the ISPO (2018) that low levels of access to prosthetic and orthotic services is hampered by a broad lack of understanding of the benefits and need for these services. The World Health Organization thus encourages countries to have accurate statistics on Orthotic & Prosthetic devices needed so as to be able to estimate the number of professionals needed. Without such data, not only is it difficult to measure the extent of shortages, demand and need from national population projections but we are also not able to present data on current utilization. Accurate data on the national need for prosthetics and orthotics are rarely available, however, information on how many people require services, of what type and where they live is indispensable for planning and developing country-wide services for all [7].

This study shows the slow pace of transformation towards making rehabilitation services available and accessible despite the calls for decentralization and equitable distribution of human resources in healthcare, particularly when it comes to rehabilitation personnel. While the history of apartheid segregation played a role in unequal distribution of this rehabilitation workforce, especially between the rural and urban developments, it also played a significant role on limited availability and access to training opportunities for a diverse group of prosthetists and orthotists. The impact of this is felt mostly in the unequal distribution and service provision which is frequently available only in major cities and towns and for those who can access private sector funds while the poor, and persons in isolated populations especially in rural areas are dependent on the under-resourced public sector. Services for the poor are usually provided by charitable organizations which often offer assistive technology products of arguably quality that pose various risks to secondary preventable impairments and deformities. Additionally, prosthetics and orthotics services are frequently perceived as an expense rather than an investment [12].

Trained personnel in prosthetic and orthotic services, are significantly lacking worldwide and in Africa in particular [28,29]. This subsequently influences the provision of high-quality prostheses and orthoses as it depends on the availability of competent, adequately trained practitioners and other health care personnel [7]. It is important that the education and training offered is of high-quality [30] as the trained prosthetic and orthotic professionals often become managers and trainers involved in the in-service training of those who are providing the required services [31]. The rise in HR for rehabilitation we see now have been facilitated by an increase in local training. The South African government has taken initiatives to open up training programmes in two universities namely; Durban University of Technology (DUT) in KwaZulu Natal and Tshwane University of Technology (TUT) in Gauteng. The introduction of these training programmes gave rise to a rapid increase in numbers of P/Os human resources. For instance, this may explain the rise in the number of practitioners which happened in 2017 in KZN with 50 professionals registering with HPCSA. Similarly with the Gauteng province which has been enjoying higher numbers of P/Os due to the fact that, for years, the P/O programme was only available at TUT located in the province. Laudable as these steps are, however, considerable more still needs to be done in terms of increasing the number of P/Os to be able to reach the majority population in the impoverished provinces. The training standards developed by the Global Standards for Prosthetics and Orthotics, recognize the importance of having competent professionals, who are trained appropriately within a multidisciplinary team for complex cases [32]. It also highlights that training should be aligned with both national as well as international education standards and acknowledges continuing professional development as a compulsory activity [32]. These measures need to be accompanied by policy plans for prosthetics and orthotics, rehabilitation and assistive technology to inform financing as these services are frequently not budgeted for in national health and social insurance systems.

## Conclusion

Strengthening rehabilitation requires better information on human resources at national and provincial levels. This study shows unequal spatial distribution trends of P/Os between provinces which could be accounted for by the country’s history and its slow pace of transformation particularly with improving rehabilitation services. A majority of workforce is situated in the urban areas while the demand in rural contexts remains high and unfulfilled. Interestingly provinces with a higher density of P/Os have lower disability prevalence while those with lower density have higher prevalence. Addressing these existing shortages will thus not only widen access to P/Os service delivery but will also ensure that adequate infrastructure to provide these services is motivated and planned for. The study presents a compelling case for prioritization and strengthening of this workforce for effective universal health coverage for persons with disabilities.

A particular strength of this study is that it highlights the existing shortages of P/Os and a gap in data recording in South Africa. This is a timely crucial step for addressing and guiding activities in line with the intentions of the FSDR but also contributes in shaping the planning and financing for UHC of rehabilitation services. In order to implement the FSDR, we argue that both NHI and UHC should prioritize and strengthen disability and rehabilitation workforce as a way of upscaling rehabilitation services and access. The provincial breakdown is particularly crucial to inform the varying budgets and resource allocations required for policy implementation at provincial levels.

There is an urgent need to recognize the importance of P/Os as they play a crucial role in the rehabilitation of persons with disabilities thus enabling their participation in various aspects of life. The first step is the recognition of the urgent need for workforce planning which needs to focus on recruiting and retaining appropriate service providers as well as recognize and prioritize the workforce required in prosthetics and orthotics services at all levels of healthcare. We recommend national and provincial stakeholders to ensure a workforce that has local context but is flexible enough to adapt to changing conditions. This requires an upscaling of training by the higher education institutions. Government should also move beyond policy and include planning, implementation and monitoring by taking a leadership role in bringing stakeholders together to develop a national approach for prosthetics and orthotics services. Though SAOPA and MOPASA exist, there is a need for a national prosthetics and orthotics committee or similar entity to lead a national guiding framework for workforce planning, monitoring and evaluation.

While this study provides much needed data on the current human resource status of P/Os to catalyse efforts towards improving future supply and demand in line with the intentions of the FSDR, it is, however, not without limitations. One limitation relates to data sources. Unfortunately, the data currently available on P/Os in South Africa is limited. The HPCSA database used in this study offers the most complete set of information currently being collected, but as has been seen, leaves many questions unanswered. Data on P/Os employed in the public health sector (including funded posts and vacancies) is not consistently collected or made available by the various provincial health departments, although efforts are being made to address this. Persal, the human resources system used in the public sector, is not inclusive of P/Os in private sector. At present, we also know little about where of P/Os currently registered with the HPCSA but not employed by the Department of Health are working or practising in South Africa. While SAOPA collects some of this information from its private sector members, membership is not compulsory nor universal and we were unable to access the latest registration list from SAOPA. Finally, this study used HPCSA data up to 2018, and therefore does not account for changes in public sector health vacancies and other trends since that date.

## List of abbreviations

B.Sc: Bachelor of Science
BTech: Bachelor of Technology
DUT: Durban University of Technology
FSDR: Framework and Strategy for Disability and Rehabilitation
HPCSA: Health Professions Council of South Africa
HREC: Health Research Ethics Committee
HRH: Human Resources for Health
ISPO: International Society of Prosthetics and Orthotics
MOPASA: Medical Orthotists & Prosthetists Association of South Africa
NDOH: National Department of Health
NGO: Non-governmental Sector
O&P: Orthotic & Prosthetic
P/Os: Prosthetist/Orthotist
PERSAL: Personal and Salaries System
PHC: Primary Health Care
SA: South Africa
SAOPA: South African Orthotic and Prosthetic Association
SDGs: Sustainable Development Goals
SPSS: Statistical Package for the Social Sciences
TUT: Tshwane University of Technology
UHC: Universal Health Coverage
UN: United Nations
WHO: World Health Organization
WSU: Walter Sisulu University
YLL: Years of Life Lost

## Paper context

This article reviews South Africa’s preparedness with respect to its human resources for health related to prosthetic and/or orthotic services. In this article, we have attempted to attribute for demographic profiling of P/Os highlighting geospatial inequity which contributes to inaccessibility of services. The paper calls for greater prominence of prosthetic and/or orthotic services on South Africa’s health agendas campaigning for rehabilitation and universal health coverage.
